# Supplementary material for: Mapping the evolution of fertility support policies in China: A content and instrumental analysis
Source: PLoS One. 2025 Oct 9;20(10):e0332137. doi: 10.1371/journal.pone.0332137 (PMC12510515; doi:10.1371/journal.pone.0332137)
Supplement: S1 Appendix — (ZIP) [file pone.0332137.s001.zip › S1 Appendix. 226 original policy documents/73-国务院关于落实《政府工作报告》重点工作部门分工的意见(2020)(FBM-CLI-2-343023).doc]

国务院关于落实《政府工作报告》重点工作部门分工的意见(2020)

**发布部门：** [国务院](https://www.pkulaw.com/law/chl?Aggs.IssueDepartment=5)

**发文字号：**国发〔2020〕6号

**发布日期：**2020.06.06

**实施日期：**2020.06.06

**时效性：** [现行有效](https://www.pkulaw.com/law/chl?Aggs.TimelinessDic=01)

**效力级别：** [国务院规范性文件](https://www.pkulaw.com/law/chl?Aggs.EffectivenessDic=XC0203)

**法规类别：** [机关工作综合规定](https://www.pkulaw.com/law/chl?Aggs.Category=00301) [营商环境优化](https://dobusiness.pkulaw.com/)

国务院关于落实《政府工作报告》重点工作部门分工的意见
（国发〔2020〕6号）

各省、自治区、直辖市人民政府，国务院各部委、各直属机构：
　　为全面贯彻党的十九大和十九届二中、三中、四中全会精神，深入落实中央经济工作会议精神和十三届全国人大三次会议通过的《政府工作报告》部署，做好今年政府工作，实现经济社会发展目标任务，现就《政府工作报告》确定的重点工作，提出部门分工意见如下：
　　**一、**落实2020年发展主要目标和下一阶段工作总体部署
　　（一）总体要求和主要预期目标。
　　1.在以习近平同志为核心的党中央坚强领导下，以习近平新时代中国特色社会主义思想为指导，全面贯彻党的十九大和十九届二中、三中、四中全会精神，坚决贯彻党的基本理论、基本路线、基本方略，增强“四个意识”、坚定“四个自信”、做到“两个维护”，紧扣全面建成小康社会目标任务，统筹推进疫情防控和经济社会发展工作，在疫情防控常态化前提下，坚持稳中求进工作总基调，坚持新发展理念，坚持以供给侧结构性改革为主线，坚持以改革开放为动力推动高质量发展，坚决打好三大攻坚战，加大“六稳”工作力度，保居民就业、保基本民生、保市场主体、保粮食能源安全、保产业链供应链稳定、保基层运转，坚定实施扩大内需战略，维护经济发展和社会稳定大局，确保完成决战决胜脱贫攻坚目标任务，全面建成小康社会。（国务院各部门分别负责，年内持续推进）
　　2.优先稳就业保民生，坚决打赢脱贫攻坚战，努力实现全面建成小康社会目标任务；城镇新增就业900万人以上，城镇调查失业率6%左右，城镇登记失业率5.5%左右；居民消费价格涨幅3.5%左右；进出口促稳提质，国际收支基本平衡；居民收入增长与经济增长基本同步；现行标准下农村贫困人口全部脱贫、贫困县全部摘帽；重大金融风险有效防控；单位国内生产总值能耗和主要污染物排放量继续下降，努力完成“十三五”规划目标任务。（国家发展改革委、财政部、人力资源社会保障部、生态环境部、商务部、人民银行、海关总署、银保监会、证监会、国家外汇局、国务院扶贫办等按职责分工负责，12月底前完成）
　　（二）积极的财政政策要更加积极有为。
　　3.今年赤字率拟按3.6%以上安排，财政赤字规模比去年增加1万亿元，同时发行1万亿元抗疫特别国债。上述2万亿元全部转给地方，建立特殊转移支付机制，资金直达市县基层、直接惠企利民，主要用于保就业、保基本民生、保市场主体，包括支持减税降费、减租降息、扩大消费和投资等，强化公共财政属性，决不允许截留挪用。要大力优化财政支出结构，基本民生支出只增不减，重点领域支出要切实保障，一般性支出要坚决压减，严禁新建楼堂馆所，严禁铺张浪费。各级政府必须真正过紧日子，中央政府要带头，中央本级支出安排负增长，其中非急需非刚性支出压减50%以上。各类结余、沉淀资金要应收尽收、重新安排。要大力提质增效，各项支出务必精打细算，一定要把每一笔钱都用在刀刃上、紧要处，一定要让市场主体和人民群众有真真切切的感受。（财政部牵头，国家发展改革委、人力资源社会保障部、人民银行、税务总局等按职责分工负责，12月底前完成）
　　（三）稳健的货币政策要更加灵活适度。
　　4.综合运用降准降息、再贷款等手段，引导广义货币供应量和社会融资规模增速明显高于去年。保持人民币汇率在合理均衡水平上基本稳定。（人民银行、国家外汇局牵头，年内持续推进）创新直达实体经济的货币政策工具，务必推动企业便利获得贷款，推动利率持续下行。（人民银行、银保监会、国家发展改革委、财政部等按职责分工负责，年内持续推进）
　　（四）就业优先政策要全面强化。
　　5.财政、货币和投资等政策要聚力支持稳就业。努力稳定现有就业，积极增加新的就业，促进失业人员再就业。各地要清理取消对就业的不合理限制，促就业举措要应出尽出，拓岗位办法要能用尽用。（人力资源社会保障部、国家发展改革委、财政部、人民银行等按职责分工负责，年内持续推进）
　　（五）继续打好三大攻坚战。
　　6.脱贫是全面建成小康社会必须完成的硬任务，要坚持现行脱贫标准，增加扶贫投入，强化扶贫举措落实，确保剩余贫困人口全部脱贫，健全和执行好返贫人口监测帮扶机制，巩固脱贫成果。（国务院扶贫办牵头，年内持续推进）
　　7.要打好蓝天、碧水、净土保卫战，实现污染防治攻坚战阶段性目标。（生态环境部牵头，年内持续推进）
　　8.加强金融等领域重大风险防控，坚决守住不发生系统性风险底线。（人民银行、银保监会、证监会、国家发展改革委、财政部、国家外汇局等按职责分工负责，年内持续推进）

　　**二、**加大宏观政策实施力度，着力稳企业保就业
　　（六）加大减税降费力度。
　　9.强化阶段性政策，与制度性安排相结合，放水养鱼，助力市场主体纾困发展。继续执行去年出台的下调增值税税率和企业养老保险费率政策，新增减税降费约5000亿元。前期出台6月前到期的减税降费政策，包括免征中小微企业养老、失业和工伤保险单位缴费，减免小规模纳税人增值税，免征公共交通运输、餐饮住宿、旅游娱乐、文化体育等服务增值税，减免民航发展基金、港口建设费，执行期限全部延长到今年年底。小微企业、个体工商户所得税缴纳一律延缓到明年。预计全年为企业新增减负超过2.5万亿元。要坚决把减税降费政策落到企业，尽力帮助企业特别是中小微企业、个体工商户渡过难关。（财政部、国家发展改革委、工业和信息化部、人力资源社会保障部、住房城乡建设部、交通运输部、商务部、文化和旅游部、税务总局、市场监管总局、国家医保局、中国民航局等按职责分工负责，年内持续推进）
　　（七）推动降低企业生产经营成本。
　　10.降低工商业电价5%政策延长到今年年底。宽带和专线平均资费降低15%。减免国有房产租金，鼓励各类业主减免或缓收房租，并予政策支持。坚决整治涉企违规收费。（国家发展改革委、工业和信息化部、财政部、住房城乡建设部、国务院国资委、市场监管总局等按职责分工负责，6月底前出台相关政策，年内持续推进）
　　（八）强化对稳企业的金融支持。
　　11.中小微企业贷款延期还本付息政策再延长至明年3月底，对普惠型小微企业贷款应延尽延，对其他困难企业贷款协商延期。（人民银行、银保监会牵头，6月底前出台相关政策，年内持续推进）完善考核激励机制，鼓励银行敢贷、愿贷、能贷，大幅增加小微企业信用贷、首贷、无还本续贷，利用金融科技和大数据降低服务成本，提高服务精准性。（人民银行、银保监会、国家发展改革委牵头，6月底前出台相关政策，年内持续推进）大幅拓展政府性融资担保覆盖面并明显降低费率。（财政部、银保监会牵头，年内持续推进）大型商业银行普惠型小微企业贷款增速要高于40%。（银保监会、人民银行牵头，12月底前完成）促进涉企信用信息共享。（国家发展改革委、人民银行、市场监管总局等按职责分工负责，年内持续推进）支持企业扩大债券融资。（人民银行、国家发展改革委、证监会等按职责分工负责，8月底前出台相关政策，年内持续推进）加强监管，防止资金“空转”套利，打击恶意逃废债。金融机构与贷款企业共生共荣，鼓励银行合理让利。（国家发展改革委、财政部、人民银行、银保监会、证监会等按职责分工负责，年内持续推进）为保市场主体，一定要让中小微企业贷款可获得性明显提高，一定要让综合融资成本明显下降。（人民银行、银保监会、财政部等按职责分工负责，适时出台相关政策，年内持续推进）
　　（九）千方百计稳定和扩大就业。
　　12.加强对重点行业、重点群体就业支持。（人力资源社会保障部牵头，年内持续推进）今年高校毕业生达874万人，要促进市场化社会化就业，高校和属地政府都要提供不断线的就业服务，扩大基层服务项目招聘。（人力资源社会保障部、教育部、财政部等按职责分工负责，年内持续推进）做好退役军人安置和就业保障。（退役军人部牵头，年内持续推进）实行农民工在就业地平等享受就业服务政策。（人力资源社会保障部牵头，年内持续推进）帮扶残疾人、零就业家庭等困难群体就业。（人力资源社会保障部、中国残联等按职责分工负责，年内持续推进）今年对低收入人员实行社保费自愿缓缴政策，涉及就业的行政事业性收费全部取消，合理设定流动摊贩经营场所。（人力资源社会保障部、财政部、国家发展改革委、住房城乡建设部、税务总局等按职责分工负责，7月底前出台相关政策）资助以训稳岗拓岗，加强面向市场的技能培训，鼓励以工代训，共建共享生产性实训基地，今明两年职业技能培训3500万人次以上，高职院校扩招200万人，要使更多劳动者长技能、好就业。（人力资源社会保障部、教育部、国家发展改革委、财政部等按职责分工负责，12月底前完成当年任务）

　　**三、**依靠改革激发市场主体活力，增强发展新动能
　　（十）深化“放管服”改革。
　　13.在常态化疫情防控下，要调整措施、简化手续，促进全面复工复产、复市复业。（国家发展改革委、国务院办公厅牵头，有关部门按职责分工负责，年内持续推进）推动更多服务事项一网通办。（国务院办公厅牵头，有关部门按职责分工负责，年内持续推进）做到企业开办全程网上办理。放宽小微企业、个体工商户登记经营场所限制，便利各类创业者注册经营、及时享受扶持政策。（市场监管总局牵头，12月底前完成）支持大中小企业融通发展。（工业和信息化部牵头，年内持续推进）完善社会信用体系。（国家发展改革委、人民银行牵头，适时出台相关政策，年内持续推进）以公正监管维护公平竞争，持续打造市场化、法治化、国际化营商环境。（国务院办公厅、国家发展改革委、司法部、财政部、住房城乡建设部、市场监管总局等按职责分工负责，年内持续推进）
　　（十一）推进要素市场化配置改革。
　　14.推动中小银行补充资本和完善治理，更好服务中小微企业。（银保监会、财政部、人民银行、证监会等按职责分工负责，适时出台相关政策，年内持续推进）改革创业板并试点注册制，发展多层次资本市场。（证监会牵头，6月底前出台相关政策，年内落地）强化保险保障功能。（银保监会牵头，年内持续推进）赋予省级政府建设用地更大自主权。（自然资源部牵头，6月底前完成）促进人才流动。（人力资源社会保障部牵头，年内持续推进）培育技术和数据市场，激活各类要素潜能。（国家发展改革委、科技部、工业和信息化部等按职责分工负责，年内持续推进）
　　（十二）提升国资国企改革成效。
　　15.实施国企改革三年行动。（国务院国资委牵头，10月底前出台相关政策）健全现代企业制度，完善国资监管体制。（国务院国资委、财政部等按职责分工负责，年内持续推进）深化混合所有制改革。（国家发展改革委、国务院国资委牵头，年内持续推进）基本完成剥离办社会职能和解决历史遗留问题。（国务院国资委牵头，12月底前完成）国企要聚焦主责主业，健全市场化经营机制，提高核心竞争力。（国务院国资委牵头，年内持续推进）
　　（十三）优化民营经济发展环境。
　　16.保障民营企业平等获取生产要素和政策支持，清理废除与企业性质挂钩的不合理规定。构建亲清政商关系，促进非公有制经济健康发展。（国家发展改革委、工业和信息化部、财政部、市场监管总局、全国工商联等按职责分工负责，年内持续推进）限期完成清偿政府机构、国有企业拖欠民营和中小企业款项的任务。（工业和信息化部牵头，有关部门按职责分工负责，逐月调度通报清欠情况）
　　（十四）推动制造业升级和新兴产业发展。
　　17.支持制造业高质量发展。（工业和信息化部、国家发展改革委等按职责分工负责，年内持续推进）大幅增加制造业中长期贷款。（人民银行、银保监会、国家发展改革委牵头，年内持续推进）发展工业互联网，推进智能制造，培育新兴产业集群。发展研发设计、现代物流、检验检测认证等生产性服务业。电商网购、在线服务等新业态在抗疫中发挥了重要作用，要继续出台支持政策，全面推进“互联网＋”，打造数字经济新优势。（国家发展改革委、工业和信息化部、商务部、国家邮政局等按职责分工负责，9月底前出台相关政策，年内持续推进）
　　（十五）提高科技创新支撑能力。
　　18.稳定支持基础研究和应用基础研究，引导企业增加研发投入，促进产学研融通创新。加快建设国家实验室，重组国家重点实验室体系，发展社会研发机构，加强关键核心技术攻关。发展民生科技。深化国际科技合作。改革科技成果转化机制，畅通创新链，营造鼓励创新、宽容失败的科研环境。实行重点项目攻关“揭榜挂帅”，谁能干就让谁干。（科技部、国家发展改革委、工业和信息化部、教育部、财政部、外交部、国家卫生健康委、农业农村部、国务院国资委、中科院、工程院、国家能源局、国家国防科工局等按职责分工负责，年内持续推进）加强知识产权保护。（国家知识产权局牵头，年内持续推进）
　　（十六）深入推进大众创业万众创新。
　　19.发展创业投资和股权投资，增加创业担保贷款。（国家发展改革委、财政部、人力资源社会保障部、人民银行、银保监会、证监会等按职责分工负责，年内持续推进）深化新一轮全面创新改革试验。（国家发展改革委、科技部牵头，年内持续推进）新建一批双创示范基地。（国家发展改革委牵头，年内持续推进）坚持包容审慎监管，发展平台经济、共享经济，更大激发社会创造力。（国务院办公厅、国家发展改革委、科技部、工业和信息化部、商务部、市场监管总局等按职责分工负责，年内持续推进）

　　**四、**实施扩大内需战略，推动经济发展方式加快转变
　　（十七）推动消费回升。
　　20.通过稳就业促增收保民生，提高居民消费意愿和能力。（国家发展改革委、人力资源社会保障部、民政部、商务部、国家医保局等按职责分工负责，年内持续推进）支持餐饮、商场、文化、旅游、家政等生活服务业恢复发展，推动线上线下融合。促进汽车消费，大力解决停车难问题。发展养老、托幼服务。发展大健康产业。改造提升步行街。支持电商、快递进农村，拓展农村消费。要多措并举扩消费，适应群众多元化需求。（国家发展改革委、教育部、科技部、工业和信息化部、民政部、财政部、住房城乡建设部、农业农村部、商务部、文化和旅游部、国家卫生健康委、市场监管总局、体育总局、国家医保局、国家邮政局、国家中医药局、国家药监局、全国老龄办、全国妇联等按职责分工负责，年内持续推进）
　　（十八）扩大有效投资。
　　21.今年拟安排地方政府专项债券3.75万亿元，比去年增加1.6万亿元，提高专项债券可用作项目资本金的比例，中央预算内投资安排6000亿元。重点支持既促消费惠民生又调结构增后劲的“两新一重”建设，主要是：加强新型基础设施建设，发展新一代信息网络，拓展5G应用，建设数据中心，增加充电桩、换电站等设施，推广新能源汽车，激发新消费需求、助力产业升级。加强新型城镇化建设，大力提升县城公共设施和服务能力，以适应农民日益增加的到县城就业安家需求。新开工改造城镇老旧小区3.9万个，支持管网改造、加装电梯等，发展居家养老、用餐、保洁等多样社区服务。加强交通、水利等重大工程建设。增加国家铁路建设资本金1000亿元。健全市场化投融资机制，支持民营企业平等参与。要优选项目，不留后遗症，让投资持续发挥效益。（国家发展改革委、财政部、工业和信息化部、住房城乡建设部、交通运输部、水利部、农业农村部、民政部、商务部、国家卫生健康委、人民银行、银保监会、国家能源局、中国国家铁路集团有限公司、开发银行、进出口银行、农业发展银行等按职责分工负责，年内持续推进）
　　（十九）深入推进新型城镇化。
　　22.发挥中心城市和城市群综合带动作用，培育产业、增加就业。（国家发展改革委牵头，年内持续推进）坚持房子是用来住的、不是用来炒的定位，因城施策，促进房地产市场平稳健康发展。完善便民、无障碍设施，让城市更宜业宜居。（住房城乡建设部牵头，年内持续推进）
　　（二十）加快落实区域发展战略。
　　23.继续推动西部大开发、东北全面振兴、中部地区崛起、东部率先发展。深入推进京津冀协同发展、粤港澳大湾区建设、长三角一体化发展。推进长江经济带共抓大保护。编制黄河流域生态保护和高质量发展规划纲要。推动成渝地区双城经济圈建设。促进革命老区、民族地区、边疆地区、贫困地区加快发展。发展海洋经济。（国家发展改革委牵头，年内持续推进）
　　24.实施好支持湖北发展一揽子政策，支持保就业、保民生、保运转，促进经济社会秩序全面恢复。（国家发展改革委牵头，年内持续推进）
　　（二十一）提高生态环境治理成效。
　　25.突出依法、科学、精准治污。深化重点地区大气污染治理攻坚。（生态环境部牵头，年内持续推进）加强污水、垃圾处置设施建设，推进生活垃圾分类。（国家发展改革委、住房城乡建设部、生态环境部等按职责分工负责，年内持续推进）加快人口密集区危化品生产企业搬迁改造。（工业和信息化部牵头，年内持续推进）壮大节能环保产业。（国家发展改革委、工业和信息化部、生态环境部等按职责分工负责，年内持续推进）
　　26.严惩非法捕杀、交易、食用野生动物行为。（国家林草局、农业农村部、市场监管总局、公安部、海关总署等按职责分工负责，年内持续推进）实施重要生态系统保护和修复重大工程，促进生态文明建设。（国家发展改革委、财政部、自然资源部、生态环境部、国家林草局等按职责分工负责，年内持续推进）
　　（二十二）保障能源安全。
　　27.推动煤炭清洁高效利用，发展可再生能源，完善石油、天然气、电力产供销体系，提升能源储备能力。（国家发展改革委、财政部、国家能源局牵头，年内持续推进）

　　**五、**确保实现脱贫攻坚目标，促进农业丰收农民增收
　　（二十三）坚决打赢脱贫攻坚战。
　　28.加大剩余贫困县和贫困村攻坚力度，对外出务工劳动力，要在就业地稳岗就业。开展消费扶贫行动，支持扶贫产业恢复发展。继续执行对摘帽县的主要扶持政策。接续推进脱贫与乡村振兴有效衔接，全力让脱贫群众迈向富裕。（国务院扶贫办、国家发展改革委、人力资源社会保障部、农业农村部、人民银行、开发银行、农业发展银行等按职责分工负责，年内持续推进）加强易地扶贫搬迁后续扶持。（国家发展改革委、国务院扶贫办牵头，年内持续推进）深化东西部扶贫协作和中央单位定点扶贫。（国务院扶贫办牵头，年内持续推进）强化对特殊贫困人口兜底保障。（民政部牵头，年内持续推进）搞好脱贫攻坚普查。（国家统计局、国务院扶贫办牵头，7月开始对第1批普查对象进行现场登记）
　　（二十四）着力抓好农业生产。
　　29.稳定粮食播种面积和产量，提高复种指数，提高稻谷最低收购价，增加产粮大县奖励，大力防治重大病虫害。支持大豆等油料生产。（农业农村部、国家发展改革委、财政部等按职责分工负责，12月底前完成）惩处违法违规侵占耕地行为。（自然资源部、农业农村部等按职责分工负责，年内持续推进）新建高标准农田8000万亩。培育推广优良品种。（农业农村部牵头，12月底前完成）完善农机补贴政策。（农业农村部、财政部等按职责分工负责，年内持续推进）深化农村改革。（农业农村部牵头，年内持续推进）加强非洲猪瘟等疫病防控，恢复生猪生产，发展畜禽水产养殖。（农业农村部牵头，年内持续推进）健全农产品流通体系。（商务部、国家发展改革委、农业农村部等按职责分工负责，年内持续推进）压实“米袋子”省长负责制和“菜篮子”市长负责制。14亿中国人的饭碗，务必牢牢端在自己手中。（国家发展改革委、农业农村部、国家粮食和储备局等按职责分工负责，12月底前完成）
　　（二十五）拓展农民就业增收渠道。
　　30.支持农民就近就业创业，促进一二三产业融合发展，扩大以工代赈规模，让返乡农民工能打工、有收入。加强农民职业技能培训。依法根治拖欠农民工工资问题。（国家发展改革委、财政部、人力资源社会保障部、农业农村部等按职责分工负责，年内持续推进）扶持适度规模经营主体，加强农户社会化服务。（农业农村部牵头，年内持续推进）支持农产品深加工。（工业和信息化部、农业农村部等按职责分工负责，年内持续推进）完善乡村产业发展用地保障政策。（自然资源部、农业农村部牵头，年内持续推进）增强集体经济实力。（农业农村部牵头，年内持续推进）增加专项债券投入，支持现代农业设施、饮水安全工程和人居环境整治，持续改善农民生产生活条件。（国家发展改革委、财政部、农业农村部、水利部、住房城乡建设部、生态环境部、农业发展银行等按职责分工负责，12月底前完成）

　　**六、**推进更高水平对外开放，稳住外贸外资基本盘
　　（二十六）稳定产业链供应链。
　　31.面对外部环境变化，要坚定不移扩大对外开放，稳定产业链供应链，以开放促改革促发展。（国家发展改革委、商务部牵头，年内持续推进）
　　（二十七）促进外贸基本稳定。
　　32.围绕支持企业增订单稳岗位保就业，加大信贷投放，扩大出口信用保险覆盖面，降低进出口合规成本，支持出口产品转内销。（商务部、财政部、人民银行、银保监会、进出口银行等按职责分工负责，6月底前出台相关政策，年内持续推进）加快跨境电商等新业态发展，提升国际货运能力。（商务部、国家发展改革委、交通运输部、海关总署、国家邮政局等按职责分工负责，9月底前出台相关政策，年内持续推进）推进新一轮服务贸易创新发展试点。（商务部牵头，8月底前完成）筹办好第三届进博会，积极扩大进口，发展更高水平面向世界的大市场。（商务部牵头，11月底前完成）
　　（二十八）积极利用外资。
　　33.大幅缩减外资准入负面清单。（国家发展改革委、商务部等按职责分工负责，6月底前完成）出台跨境服务贸易负面清单。（商务部牵头，12月底前完成）深化经济特区改革开放。（国家发展改革委牵头，年内持续推进）赋予自贸试验区更大改革开放自主权。（商务部牵头，年内持续推进）在中西部地区增设自贸试验区、综合保税区，增加服务业扩大开放综合试点。（商务部、海关总署等按职责分工负责，12月底前完成）加快海南自由贸易港建设。（国家发展改革委牵头，商务部等有关部门按职责分工负责，年内持续推进）营造内外资企业一视同仁、公平竞争的市场环境。（国家发展改革委、商务部、市场监管总局等按职责分工负责，年内持续推进）
　　（二十九）高质量共建“一带一路”。
　　34.坚持共商共建共享，遵循市场原则和国际通行规则，发挥企业主体作用，开展互惠互利合作。引导对外投资健康发展。（国家发展改革委、商务部、外交部等按职责分工负责，年内持续推进）
　　（三十）推动贸易和投资自由化便利化。
　　35.坚定维护多边贸易体制，积极参与世贸组织改革。推动签署区域全面经济伙伴关系协定，推进中日韩等自贸谈判。（商务部牵头，年内持续推进）共同落实中美第一阶段经贸协议。（财政部、国家发展改革委、农业农村部、商务部、人民银行、海关总署等按职责分工负责，年内持续推进）致力于加强与各国经贸合作，实现互利共赢。（商务部牵头，年内持续推进）

　　**七、**围绕保障和改善民生，推动社会事业改革发展
　　（三十一）加强公共卫生体系建设。
　　36.坚持生命至上，改革疾病预防控制体制，加强传染病防治能力建设，完善传染病直报和预警系统，坚持及时公开透明发布疫情信息。（国家卫生健康委牵头，12月底前出台相关政策）用好抗疫特别国债，加大疫苗、药物和快速检测技术研发投入，增加防疫救治医疗设施，增加移动实验室，强化应急物资保障，强化基层卫生防疫。加快公共卫生人才队伍建设。（国家发展改革委、教育部、科技部、工业和信息化部、财政部、人力资源社会保障部、交通运输部、商务部、国家卫生健康委、市场监管总局、国家中医药局、国家药监局等按职责分工负责，年内持续推进）深入开展爱国卫生运动。普及卫生健康知识，倡导健康文明生活方式。（国家卫生健康委牵头，教育部、民政部、生态环境部、住房城乡建设部、农业农村部、市场监管总局等按职责分工负责，10月底前出台相关政策）要大幅提升防控能力，坚决防止疫情反弹，坚决守护人民健康。（国家卫生健康委牵头，年内持续推进）
　　（三十二）提高基本医疗服务水平。
　　37.居民医保人均财政补助标准增加30元。（财政部、国家医保局牵头，7月底前出台相关政策）开展门诊费用跨省直接结算试点。（国家医保局牵头，年内持续推进）对受疫情影响的医疗机构给予扶持。（国家发展改革委、财政部、人力资源社会保障部、国家卫生健康委、国家医保局等按职责分工负责，年内持续推进）深化公立医院综合改革。（国家卫生健康委、财政部牵头，12月底前出台相关政策）建设区域医疗中心。（国家发展改革委、国家卫生健康委、国家中医药局牵头，年内持续推进）发展“互联网＋医疗健康”。提高城乡社区医疗服务能力。推进分级诊疗。构建和谐医患关系。（国家卫生健康委牵头，年内持续推进）促进中医药振兴发展，加强中西医结合。（国家卫生健康委、国家中医药局、国家发展改革委牵头，教育部、科技部、财政部、农业农村部、国家医保局、国家药监局等按职责分工负责，7月底前出台相关政策，年内持续推进）严格食品药品监管，确保安全。（市场监管总局、国家药监局、海关总署等按职责分工负责，年内持续推进）
　　（三十三）推动教育公平发展和质量提升。
　　38.坚持立德树人。有序组织中小学教育教学和中高考工作。加强乡镇寄宿制学校、乡村小规模学校和县城学校建设。完善随迁子女义务教育入学政策。办好特殊教育、继续教育，支持和规范民办教育。发展普惠性学前教育，帮助民办幼儿园纾困。推动高等教育内涵式发展，推进一流大学和一流学科建设，支持中西部高校发展。扩大高校面向农村和贫困地区招生规模。发展职业教育。加强教师队伍建设。推进教育信息化。要稳定教育投入，优化投入结构，缩小城乡、区域、校际差距，让教育资源惠及所有家庭和孩子，让他们有更光明未来。（教育部牵头，国家发展改革委、工业和信息化部、司法部、财政部、人力资源社会保障部、中国残联等按职责分工负责，年内持续推进）
　　（三十四）加大基本民生保障力度。
　　39.上调退休人员基本养老金，提高城乡居民基础养老金最低标准。实现企业职工基本养老保险基金省级统收统支，提高中央调剂比例。全国近3亿人领取养老金，必须确保按时足额发放。扩大失业保险保障范围，将参保不足1年的农民工等失业人员都纳入常住地保障。（人力资源社会保障部、财政部牵头，7月底前出台相关政策）落实退役军人优抚政策。（退役军人部牵头，12月底前出台相关政策）做好因公殉职人员抚恤。（人力资源社会保障部、财政部、退役军人部等按职责分工负责，年内持续推进）
　　40.完善社会救助制度。扩大低保保障范围，对城乡困难家庭应保尽保，将符合条件的城镇失业和返乡人员及时纳入低保。对因灾因病因残遭遇暂时困难的人员，都要实施救助。要切实保障所有困难群众基本生活，保民生也必将助力更多失业人员再就业敢创业。（民政部牵头，财政部、人力资源社会保障部、中国残联等按职责分工负责，6月底前出台相关政策，年内持续推进）
　　（三十五）丰富群众精神文化生活。
　　41.培育和践行社会主义核心价值观，发展哲学社会科学、新闻出版、广播影视等事业。加强文物保护利用和非物质文化遗产传承。（文化和旅游部、广电总局、国家文物局等按职责分工负责，年内持续推进）加强公共文化服务，倡导全民健身和全民阅读，使全社会充满活力、向上向善。（文化和旅游部、体育总局等按职责分工负责，年内持续推进）筹办北京冬奥会、冬残奥会。（有关部门按职责分工负责，年内持续推进）
　　（三十六）加强和创新社会治理。
　　42.健全社区管理和服务机制。加强乡村治理。支持社会组织、人道救助、志愿服务、慈善事业等健康发展。保障妇女、儿童、老人、残疾人合法权益。（民政部、国务院妇儿工委办公室、全国老龄办、全国妇联、中国残联、中国红十字会总会等按职责分工负责，年内持续推进）完善信访制度，化解信访突出矛盾。（国家信访局牵头，有关部门按职责分工负责，年内持续推进）加强法律援助，及时解决群众合理诉求，妥善化解矛盾纠纷。（司法部牵头，年内持续推进）开展第七次全国人口普查。（国家统计局牵头，普查标准时点是2020年11月1日零时）加强国家安全能力建设。（安全部牵头，年内持续推进）完善社会治安防控体系，依法打击各类犯罪，建设更高水平的平安中国。（公安部牵头，年内持续推进）
　　（三十七）强化安全生产责任。
　　43.加强洪涝、火灾、地震等灾害防御，做好气象服务，提高应急管理、抢险救援和防灾减灾能力。实施安全生产专项整治。坚决遏制重特大事故发生。（应急部、工业和信息化部、公安部、自然资源部、生态环境部、住房城乡建设部、交通运输部、水利部、中国地震局、中国气象局、国家粮食和储备局、国家林草局等按职责分工负责，年内持续推进）

　　**八、**加强政府自身建设，做好“十四五”规划编制
　　（三十八）坚持依法行政，建设廉洁政府。
　　44.各级政府要自觉在思想上政治上行动上同以习近平同志为核心的党中央保持高度一致，践行以人民为中心的发展思想，落实全面从严治党要求，坚持依法行政，建设法治政府，坚持政务公开，提高治理能力。要依法接受同级人大及其常委会的监督，自觉接受人民政协的民主监督，主动接受社会和舆论监督。发挥好工会、共青团、妇联等群团组织作用。政府工作人员要自觉接受法律、监察和人民监督。加强廉洁政府建设，坚决惩治腐败。（国务院各部门分别负责，年内持续推进）强化审计监督。（审计署牵头，年内持续推进）
　　（三十九）突出求真务实，主动担当作为。
　　45.各级政府要始终坚持实事求是，牢牢把握社会主义初级阶段这个基本国情，遵循客观规律，一切从实际出发，立足办好自己的事。要大力纠治“四风”，力戒形式主义、官僚主义，把广大基层干部干事创业的手脚从形式主义的束缚中解脱出来，为担当者担当，让履职者尽责。要紧紧依靠人民群众，尊重基层首创精神，以更大力度推进改革开放，激发社会活力，凝聚亿万群众的智慧和力量。广大干部应临难不避、实干为要，凝心聚力抓发展、保民生。（国务院各部门分别负责，年内持续推进）
　　（四十）做好“十四五”规划编制工作。
　　46.今年要编制好“十四五”规划，为开启第二个百年奋斗目标新征程擘画蓝图。（国家发展改革委牵头，年内持续推进）

　　**九、**做好民族、宗教、侨务、国防、港澳台、外交工作
　　（四十一）全面贯彻党的民族政策和宗教工作基本方针，认真落实侨务政策。
　　47.要坚持和完善民族区域自治制度，支持少数民族和民族地区加快发展，铸牢中华民族共同体意识。（国家民委牵头，年内持续推进）全面贯彻党的宗教工作基本方针，发挥宗教界人士和信教群众在促进经济社会发展中的积极作用。（有关部门按职责分工负责，年内持续推进）要发挥好侨胞侨眷的独特优势，不断增强中华儿女凝聚力，同心共创辉煌。（有关部门按职责分工负责，年内持续推进）
　　（四十二）扎实推进国防和军队建设。
　　48.要深入贯彻习近平强军思想，深入贯彻新时代军事战略方针，坚持政治建军、改革强军、科技强军、人才强军、依法治军。坚持党对人民军队的绝对领导，严格落实军委主席负责制。全力加强练兵备战，坚定维护国家主权、安全、发展利益。打好军队建设发展“十三五”规划落实攻坚战，编制军队建设“十四五”规划。深化国防和军队改革，提高后勤和装备保障能力。完善国防动员体系，始终让军政军民团结坚如磐石。（有关部门按职责分工负责，年内持续推进）推动国防科技创新发展。（国家国防科工局等按职责分工负责，年内持续推进）
　　（四十三）全面准确贯彻“一国两制”、“港人治港”、“澳人治澳”、高度自治的方针。
　　49.建立健全特别行政区维护国家安全的法律制度和执行机制，落实特区政府的宪制责任。支持港澳发展经济、改善民生，更好融入国家发展大局，保持香港、澳门长期繁荣稳定。（国务院港澳办牵头，年内持续推进）
　　（四十四）坚持对台工作大政方针。
　　50.坚持一个中国原则，在“九二共识”基础上推动两岸关系和平发展。坚决反对和遏制“***”分裂行径。完善促进两岸交流合作、深化两岸融合发展、保障台湾同胞福祉的制度安排和政策措施，团结广大台湾同胞共同反对“***”、促进统一，开创民族复兴的美好未来。（有关部门按职责分工负责，年内持续推进）
　　（四十五）坚定不移走和平发展道路。
　　51.同各国携手共进，应对公共卫生危机、经济严重衰退等全球性挑战。加强防疫合作，促进世界经济稳定，推进全球治理，维护以联合国为核心的国际体系和以国际法为基础的国际秩序，推动构建人类命运共同体。坚定不移走和平发展道路，在扩大开放中深化与各国友好合作。（外交部、科技部、商务部、国家卫生健康委、国际发展合作署、国家中医药局、国家药监局等按职责分工负责，年内持续推进）
　　今年是决胜全面小康、决战脱贫攻坚的收官之年，也是实现“两个一百年”奋斗目标的历史交汇之年。我们要更加紧密地团结在以习近平同志为核心的党中央周围，高举中国特色社会主义伟大旗帜，以习近平新时代中国特色社会主义思想为指导，认真贯彻党中央、国务院决策部署，迎难而上，锐意进取，统筹推进疫情防控和经济社会发展，努力完成全年目标任务。一是加强组织领导。各部门主要负责同志要切实履职尽责，抓紧组织制定本部门贯彻实施方案，于6月20日前报国务院。要对照分工逐项建立工作台账，明确时间表、责任人和成果形式，确保各项任务有措施、可量化、能落实。二是强化协同配合。各部门要切实加大工作力度，加快工作进度，任务牵头部门和协办部门要立足大局，协同配合，形成合力，不推诿扯皮。三是注重统筹兼顾。各部门要加强形势研判，统筹国内和国际，兼顾当前和长远，坚定发展信心，增强发展动力。要深入实际、深入基层、深入群众，及时推动解决实际困难和问题，根据形势变化调整完善政策。四是抓好督查督办。各部门要增强责任感和紧迫感，主动担当、积极作为，狠抓工作落实，做好日常跟踪督办。要切实加强对各地的指导服务，减轻基层负担。各省（自治区、直辖市）人民政府要主动对接国务院有关部门，结合本地实际进一步明确工作重点，细化措施，抓好落实。国务院办公厅建立《政府工作报告》督查总台账，定期对账督办，对进度慢、办事敷衍、成效不明显的开展专项督查，对不作为、乱作为的坚决追责问责，切实推动各项重点工作落地见效。

国务院
2020年6月6日

© 北大法宝：（[www.pkulaw.com](https://www.pkulaw.com/)) 专业提供法律信息、法学知识和法律软件领域各类解决方案。北大法宝为您提供丰富的参考资料，正式引用法规条文时请与标准文本核对。
欢迎查看所有[产品和服务](https://www.pkulaw.com/helps/2.html)。法宝快讯：[如何快速找到您需要的检索结果？](https://www.pkulaw.com/helps/70.html)[法宝](https://www.pkulaw.com/helps/69.html)V6有何新特色？

扫描二维码阅读原文

**原文链接：**<https://www.pkulaw.com/chl/5bf40fc2c0b7dab1bdfb.html>
